# Violência no trabalho do policial penal: uma questão de hierarquia?

**DOI:** 10.1590/0102-311XPT154423

**Published:** 2024-02-02

**Authors:** Graziella Lage Oliveira, Adalgisa Peixoto Ribeiro

**Affiliations:** 1 Universidade Federal de Minas Gerais, Belo Horizonte, Brasil.

O trabalhador do sistema prisional brasileiro carrega consigo o estigma de lidar com os rejeitados, os bandidos perigosos. Pouco se conhece sobre a vida e atuação desses trabalhadores; no entanto, são recorrentemente qualificados como violentos, corruptos e torturadores ^1^.

Além do estigma, essa categoria profissional lida frequentemente com o assédio moral no trabalho. Definido como uma das expressões da violência, o assédio moral está relacionado a qualquer conduta abusiva, manifestada por meio de ações, palavras, gestos ou escritos que possam prejudicar a personalidade, dignidade, integridade física ou mental do trabalhador, além de ameaçar a manutenção do emprego e a deterioração do ambiente de trabalho ^2^. Inclui desqualificação, isolamento, atribuição de tarefas de menor valor com o objetivo de humilhar, indução ao erro, assédio sexual, exclusão, alterações de horários, jornadas e atividades sem prévio aviso e abusos de poder. Uma das consequências mais graves do assédio moral é o suicídio, considerado o ápice das experiências de psicoterror, hostilidade e aniquilamento psíquico ^3^. Mais que um conflito, o assédio moral no trabalho é uma forma de abuso hierárquico e de dominação ^2^.

Na perspectiva de compreender melhor o universo desses trabalhadores, Rodrigo Xavier Silva conduziu um estudo que resultou no livro *Assédio Moral: Caracterização e Incidência no Sistema Penitenciário de Minas Gerais*^4^. O livro é fruto de um estudo baseado em denúncias registradas na Diretoria de Atenção ao Servidor da Secretaria de Administração Prisional de Minas Gerais, entre 2015 e 2016. Organizado em 11 capítulos, o livro descreve os objetivos da pesquisa; fundamentação teórica sobre o assédio moral; aspectos legais da identificação, denúncia e possibilidades de punição; o sistema prisional mineiro e suas especificidades; resultados e discussões à luz da literatura da área da saúde. O estudo objetivou analisar e caracterizar as ocorrências de assédio moral e discutir os relatos a partir da legislação estadual existente.

Na fundamentação teórica, o autor discute o histórico do trabalho desde a reforma protestante até o século XXI e conceitua o termo, chamando a atenção para o adoecimento ocasionado pelo trabalho nos dias atuais. Então, introduz o papel do ambiente no adoecimento dos trabalhadores: a psicodinâmica do trabalho e a nocividade do ambiente carcerário, que é permeado por constantes tensões, ameaças de rebelião e violência e pela representação social negativa. Apresenta o conceito de assédio moral e as ações normalmente adotadas pelas vítimas face à sua ocorrência.

Sobre os aspectos legais do assédio moral, o autor indica uma lacuna na legislação no que se refere à tipificação penal do assédio moral no Direito Penal. Apresenta normativas estaduais, que especificam ações de trabalhadores e gestores diante do assédio moral nas instituições do estado, estabelecendo, entre outras, a necessidade de criação de um canal eletrônico para denúncias e de uma Comissão de Conciliação. O autor discute, ainda, as formas de manifestação do assédio moral, seus tipos, causas, consequências e formas de prevenção.

Uma seção é dedicada à discussão sobre os vínculos empregatícios no âmbito do sistema penitenciário e sua influência nas relações de trabalho. A competição velada entre servidores efetivos e contratados tende a resultar em desconfiança e hostilidade. Os vínculos precários dos contratados abrem espaço ao assédio moral, concretizado por ameaça constante de rescisão contratual; os que têm vínculos estáveis (servidores efetivos) enfrentam obstáculos para progredir na carreira. Tais desigualdades em relação ao vínculo empregatício geram um ambiente propício para o assédio moral, que atua como um elemento de sujeição e modelagem do coletivo.

A partir de 2019, com a *Emenda Constitucional nº 104/2019*^5^, os agentes penitenciários passaram a ser denominados policiais penais, responsáveis pela inspeção de celas, vigilância dos presos e controle de entrada e saída de itens. Em seu ambiente de trabalho, os policiais penais vivenciam um clima emocionalmente desafiador, responsabilizando-se por garantir um equilíbrio delicado entre presos e agentes, trabalhando sob pressão e sobrecarga. Em 2017, Minas Gerais registrou uma média de 15 presos para cada policial penal, contrariando a recomendação do Conselho Nacional de Política Criminal e Penitenciária (CNPCP) de um policial penal para cada cinco encarcerados ^1^. Embora não haja dados mais atualizados sobre a proporção de presos por agentes penais, acredita-se que a situação ainda seja bastante delicada, visto que a taxa de aprisionamento no estado, em 2022, foi de 307,38 presos a cada 100 mil habitantes ^6^.

A análise qualitativa das 68 denúncias de assédio moral, formalmente registradas entre 2015-2016 em Minas Gerais, evidenciou: condições de trabalho consideradas de extremo risco; treinamento diferenciado de acordo com o tipo de contratação; sensação de insegurança resultante da precariedade/falta de equipamentos e insumos para garantir a segurança individual e dos presos; sentimento de medo e rivalidade quase explícita entre agentes com diferentes vínculos de trabalho; e uso do assédio moral como vingança ou punição aos que identificam problemas e/ou expressam desacordo com a conduta da chefia imediata.

A relação entre a ocorrência de assédio moral e problemas de saúde é abordada no livro a partir dos afastamentos por licença médica. O autor dedicou pouco espaço de discussão sobre esse tema, o que pode ser considerado uma fragilidade. Embora não fosse o objetivo central do estudo, essa é uma discussão relevante e oportuna, já que estudos conduzidos com policiais penais destacam prevalências de transtornos mentais comuns (23,57%-33,5%), estresse (46,2%-53,7%), abuso de álcool (9,6%-88,3%) e queixas de doenças (91,6%-93,6%) ^7^. Tais problemas associam-se a piores condições do ambiente de trabalho, insultos ou situações constrangedoras e assédio moral ou sexual, tornando o assédio moral no trabalho um importante objeto de interesse da saúde coletiva, sobretudo da saúde do trabalhador.

Das 68 denúncias analisadas no livro, 85% foram classificadas como vertical descendente (praticada por superior hierárquico), 12% como horizontal (praticada entre pares) e 3% como vertical ascendente (do subordinado para a chefia). Entre as manifestações do assédio, 50% se caracterizam como deterioração proposital das condições de trabalho, 28% por atentado contra a dignidade do profissional, 13% por isolamento e recusa de comunicação e 9% por violência verbal, física ou sexual. Das 209 unidades prisionais do estado, 16% tiveram denúncias de assédio moral e apenas 21% dos casos seguiram para o desfecho da conciliação. Medo de se expor ao assediador e o sofrimento causado pela situação podem explicar os casos de desistência da denúncia (3%).

Mesmo com legislação própria sobre classificação e abordagem dos casos de assédio moral na administração pública mineira, a inexistência de previsão de punição para os agressores age como desestímulo às denúncias. O autor ressalta a subnotificação dos casos e a importância da criação de políticas públicas que invistam na atenção primária do assédio moral nas organizações penitenciárias e que fortaleçam os mecanismos de repressão com o aprimoramento dos canais de denúncia. Por fim, questiona até que ponto a gestão de pessoas e a organização do trabalho nessas instituições, marcadas por rígida hierarquia e monopólio da violência, podem estimular práticas assediadoras em lugar de promover um ambiente de trabalho digno e humano.

De forma geral, o livro cumpre seu papel de chamar a atenção para o assédio moral contra os policiais penais e abordar questões estruturantes para sua ocorrência, por exemplo, o uso do poder hierárquico, o papel dos vínculos precarizados e a dificuldade de reconhecimento e registro do assédio moral, como fatores de risco para sua ocorrência.

O livro pode ser indicado a trabalhadores do sistema penitenciário, pesquisadores e pós-graduandos com interesse no tema. Sua leitura instiga a reflexão sobre a necessidade de mais estudos que abordem os aspectos de saúde mental dos policiais penais e as consequências do assédio moral para a saúde, incluindo o uso abusivo de substâncias. Além disso, sua análise, a partir de um recorte de gênero e de vínculos empregatícios, e as demandas por atenção psicossocial e de serviços de atenção primária em saúde pelos policiais penais podem guiar a gestão no manejo dos casos.
